# An in silico to in vivo approach identifies retinoid-X receptor activating tert-butylphenols used in food contact materials

**DOI:** 10.1038/s41598-025-09244-z

**Published:** 2025-07-18

**Authors:** Brenda J. Mengeling, Azhagiya Singam Ettayapuram Ramaprasad, Martyn T. Smith, Dania Turkieh, Nicole C. Kleinstreuer, Kamel Mansouri, Kathleen A. Durkin, Michele A. La Merrill, J. David Furlow

**Affiliations:** 1 Department of Neurobiology, Physiology, and Behavior, College of Biological Sciences, University of California, Davis, CA 95616 USA; 2 Molecular Graphics and Computation Facility, College of Chemistry, University of California, Berkeley, CA 94720 USA; 3 Division of Environmental Health Sciences, School of Public Health, University of California, Berkeley, CA 94720 USA; 4 National Toxicology Program Interagency Center for the Evaluation of Alternative Toxicological Methods, National Institute of Environmental Health Sciences, Research Triangle Park, Durham, NC 27711 USA; 5 Department of Environmental Toxicology, College of Agricultural and Environmental Studies, University of California, Davis, CA 95616 USA

**Keywords:** Retinoid-X receptor, Molecular docking, Environmental exposure, Food contact chemicals, Machine learning, Artificial intelligence, New approach methods (NAMs), Biological techniques, Computational biology and bioinformatics, Developmental biology, Physiology

## Abstract

The potential for food contact chemicals to disrupt genetic programs in development and metabolism raises concerns. Nuclear receptors (NRs) control many of these programs, and the retinoid-X receptor (RXR) is a DNA-binding partner for one-third of the NRs. RXR disruption could generate adverse outcomes in several NR pathways. We used machine learning and other in silico methods to identify RXR-interacting candidates from a list of over 57,000 chemicals. Butylphenols comprised the largest, high-probability, structural group (58 compounds); several are food contact chemicals with widespread commercial use. In vitro ToxCast data suggested that bulky, aliphatic substitution at C4 of 2,6-di-tert-butylphenol facilitated RXR activation. We tested six butylphenols with increasing bulk at C4 in vivo for their ability to disrupt thyroid hormone receptor (TR) signaling, using an integrated luciferase reporter driven by TR-RXR binding and quantifiable morphological changes in a *Xenopus laevis* precocious metamorphosis assay. Three tert-butylphenols potentiated TH action at nanomolar concentrations. Molecular modeling showed the three positives formed more frequent, stable interactions with RXRα, and bulkiness at C4 increased steric complementarity with the RXR ligand-binding pocket. Our findings establish a paradigm for machine learning coupled with a convenient, in vivo validation approach to identify chemicals interacting with RXR-NR-controlled genetic pathways.

**Supplementary Information:**

The online version contains supplementary material available at 10.1038/s41598-025-09244-z.

## Introduction

One-third of vertebrate nuclear receptor (NR) transcription factors utilize the NR retinoid-X-receptor (RXR) as a binding partner to facilitate effective DNA binding^1^. Endogenous RXR ligands are tightly controlled both temporally and spatially, with recent data suggesting that RXR agonists help dictate which of its several NR binding partners it preferentially binds in a cell where multiple NRs are expressed, potentially determining biological outcomes^2,3^. In addition, RXR agonists can activate certain NR-RXR heterodimers in the absence of an agonist for the NR partner (e.g. LXR, PPARs), while also synergistically potentiating these and other NRs when that NR has bound agonist. Given the essential roles that NR-RXR dimers play in the regulation of metabolism, development, and immune function, disruption of RXR signaling by environmental chemicals has the potential to create a wide variety of adverse biological outcomes^4–7^.

Recent advances in computational toxicology hold the potential to screen large numbers of chemicals for their ability to cause different forms of toxicity. Given the vast number of chemicals humans are exposed to, future hazard identification for risk assessments will likely require integration of data from diverse sources^8^, such as in silico predictive models and high-throughput screens (HTS) accessible via the US EPA CompTox Chemicals Dashboard (https://www.epa.gov/comptox-tools/comptox-chemicals-dashboard) and NICEATM’s Integrated Chemical Environment (https://ice.ntp.niehs.nih.gov/). In this study, we utilized in silico molecular docking and machine learning models to screen over 57,000 environmental chemicals for their potential ability to interact strongly with RXR. Using clustering analysis of these in silico predictions, we chose six members of the butylphenol cluster that appear in the Food Contact Chemicals Database^9^ to test using an in vivo model for disruption of RXR action.

Previously, we have used a *Xenopus laevis* precocious metamorphosis assay to show that RXR ligands, both pharmaceutical and environmental chemicals, can potentiate the action of thyroid hormone (TH) on metamorphic pathways^10,11^. Metamorphosis of the African clawed frog, *Xenopus laevis*, is initiated and maintained through the action of TH receptors, TRs, which are NRs that heterodimerize with RXRs to regulate genetic pathways^12–15^. THs, including its most active form triiodothyronine (T3), are identical across all vertebrate taxa, and the TRs are highly conserved between *X. laevis* and humans^14^. Tadpoles at one-week post-fertilization are very uniform in developmental stage and morphology; they also readily take up hormones and chemicals through the skin from their rearing water. In addition, they haven’t yet developed a thyroid gland, and therefore, they do not yet synthesize any TH, creating a very clean model system for testing disruption of TH signaling, including disruption through the TR-binding partner RXR^16^. We used this model system to test the ability of food contact chemicals that were predicted in silico as RXR ligands to disrupt RXR function.

## Results

### In silico analysis of predicted RXR-active compounds discovered through machine learning

Using a compilation of 57,277 chemicals extended from the CoMPARA project^17^, those listed in the Food Contact Chemical Database^9^, or of interest to the State of California’s Office of Environmental Health Hazard Assessment (OEHHA), the NR-Toxpred machine learning (ML) model^18^ predicted 109 chemicals to be active at the RXR-ligand binding domain (LBD). The NR-Toxpred ML model for RXRs refined a reference chemical list from the curated Nuclear Receptor Activity database^19^, using publicly available data from PubChem, ChEMBL, and Tox21 sources. As reported previously, 80% of the active and inactive chemicals were used to train the model, and 20% were used for validation. Our RXR ML model correctly scored active RXR chemicals with Morgan Correlation Coefficient (MCC) value of 0.87 with specificity, sensitivity, and accuracy values of 100, 80, and 90 respectively for the validation set^18^. Most of the 109 predicted RXR-LBD-interacting chemicals from the 57, 277 chemical list screened here fell within the applicability domain, indicating a high confidence in these model predictions. Specifically, 104 active chemicals were considered reliable and were subjected to further in silico analysis by molecular docking and single-point free energy calculations (MM-PBSA).

**Fig. 1:**
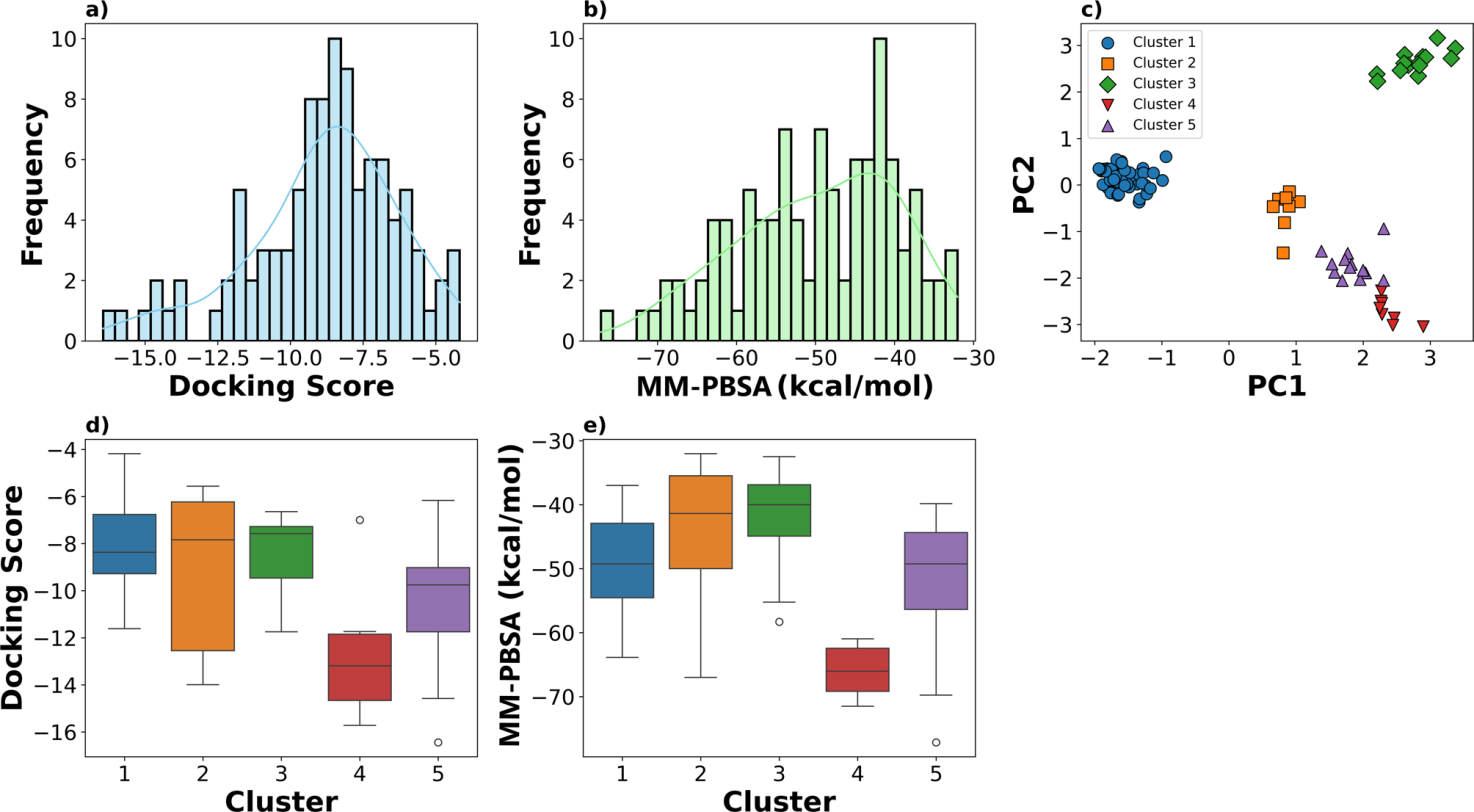
Distribution of Docking Scores and MM-PBSA Free Energy Values Across Clusters of 102 Chemicals. Negativity indicates higher affinities. (**a**) Histogram displaying the distribution of docking scores. (**b**) Histogram illustrating the distribution of MM-PBSA free energy values. (**c**) Principal Component Analysis (PCA) plot of the first two principal components (PC1 and PC2) using Morgan chemical fingerprints as features, resulting in five distinct clusters, each represented by a different color. (**d**) Box plot of the docking scores across different clusters. (**e**) Box plot of single point MM-PBSA free energy. The colors correspond to different clusters, highlighting the variation in docking scores and MM-PBSA values within and between clusters. For comparison, a known high affinity and selected RXR ligand, bexarotene (DTXSID1040619), was included in cluster 4 and has docking and MM-PBSA scores of 16.44 and -87.70, respectively. For, compound that is not known to interact with RXRs, such as tri-iodothyronine (DTXSID8023216), docking failed to generate any binding pose, consistent with its known lack of affinity for RXRs.

We employed molecular docking to determine the binding poses of these 104 chemicals in the LBD of the human RXRα (see methods section for details), using ensemble docking with multiple rigid receptor conformations obtained from the Protein Data Bank (PDB). For binding free energy calculations (MM-PBSA), we employed explicit-solvent molecular dynamics to allow for more accurate calculations with full flexibility in the protein and ligand, thereby improving the ability to extract reliable kinetic information. In addition to accounting for the dynamics of NR-ligand complex formation, there are three conserved RXR isoforms in all vertebrates. While the LBDs are particularly well conserved among the different isoforms, subtle differences may exist that would not be fully accounted for here since the vast majority of the structural data for RXRs we can use for modeling was obtained using human RXRα. The docking scores and binding free energy (MM-PBSA) results are summarized for the 104 chemicals in the supplementary information Table S1. Two of the 104 chemicals (DTXSID50205240 and DTXSID6027428) did not fit into the LBD binding pocket during docking simulations and were excluded from further testing. The distribution of docking scores and MM-PBSA free energy are shown in Fig. 1a,b, respectively. Docking scores ranged from − 16.44 to − 4.18 (mean = − 8.87), suggesting a wide range of binding affinities among the chemicals. The MM-PBSA values ranged from − 77.15 to − 32.03 (mean = − 49.79), also consistent with varying binding affinities.

To reduce the dimensionality of the dataset, we performed principal component analysis (PCA) utilizing Morgan chemical fingerprints of the remaining 102 chemicals that ML-predicted to be RXR receptor-binders, for which we also had docking and MM-PBSA scores. This produced five distinct clusters as shown in Fig. 1c. The most populated cluster, Cluster 1, contains 58 chemicals, consisting primarily of phenolic compounds heavily substituted with tert-butyl groups; several of these chemicals are common food contact chemicals (see Supplementary Information Figure S1). Cluster 1 showed consistently low variability in both docking scores (Fig. 1d) and MM-PBSA values (Fig. 1e)(Table 2). The 8 chemicals of cluster 2 are structurally diverse, but include common features such as fluorinated groups, heterocyclic rings, chlorinated compounds, carboxylic acids, ethers, and esters (see Supplementary Information Figure S2). However, members of Cluster 2 show significant variability in docking scores and MM-PBSA values (Fig. 1d,e, respectively), suggesting this diverse set of chemicals has varying binding strengths and stabilities at RXR. Cluster 3 (14 chemicals) consists primarily of coumarin derivatives, characterized by the benzopyran-2-one core structure with substitutions including methyl, ethyl, amino, and glucuronide groups (see Supplementary Information Figure S3), and Cluster 3 has higher MM-PBSA values (Fig. 1e), indicating weaker binding affinities. Cluster 4 has the lowest (highest affinity) docking scores and MM-PBSA values of any of the clusters, as expected since it is composed of retinoid and naphthenyl-derived chemicals, including various forms of the natural RXR ligand retinoic acid and its analogs (see Supplementary Information Figure S4) (Fig. 1d,e). Cluster 5 consists of 15 diverse chemicals, prominently featuring naphthalene derivatives and retinoids with sub-structural features that differ from those in cluster 4 based on their chemical fingerprints (see Supplementary Information Figure S5).

**Table 1:** Chemical information for the six food contact butylphenols.

Key	Common Name	DTXSID	CAS
2,6-DTBP	2,6-Di-tert-butylphenol	DTXSID6027052	128-39-2
BHT	Butylated hydroxytoluene	DTXSID2020216	128-37-0
4E-DTBP	2,6-Di-tert-butyl-4-ethylphenol	DTXSID0029262	4130-42-1
4sB-DTBP	4-(Butan-2-yl)-2,6-di-tert-butylphenol	DTXSID8029315	17540-75-9
TTBP	2,4,6-Tris(tert-butyl)phenol	DTXSID2021311	732-26-3
E703	2,6-Di-tert-butyl-4-[(dimethylamino)methyl]phenol	DTXSID0044997	88–27-7

DTXSID is a unique identifier derived from the US EPA DSSTox Database^20^.

### -

**Fig. 2:**
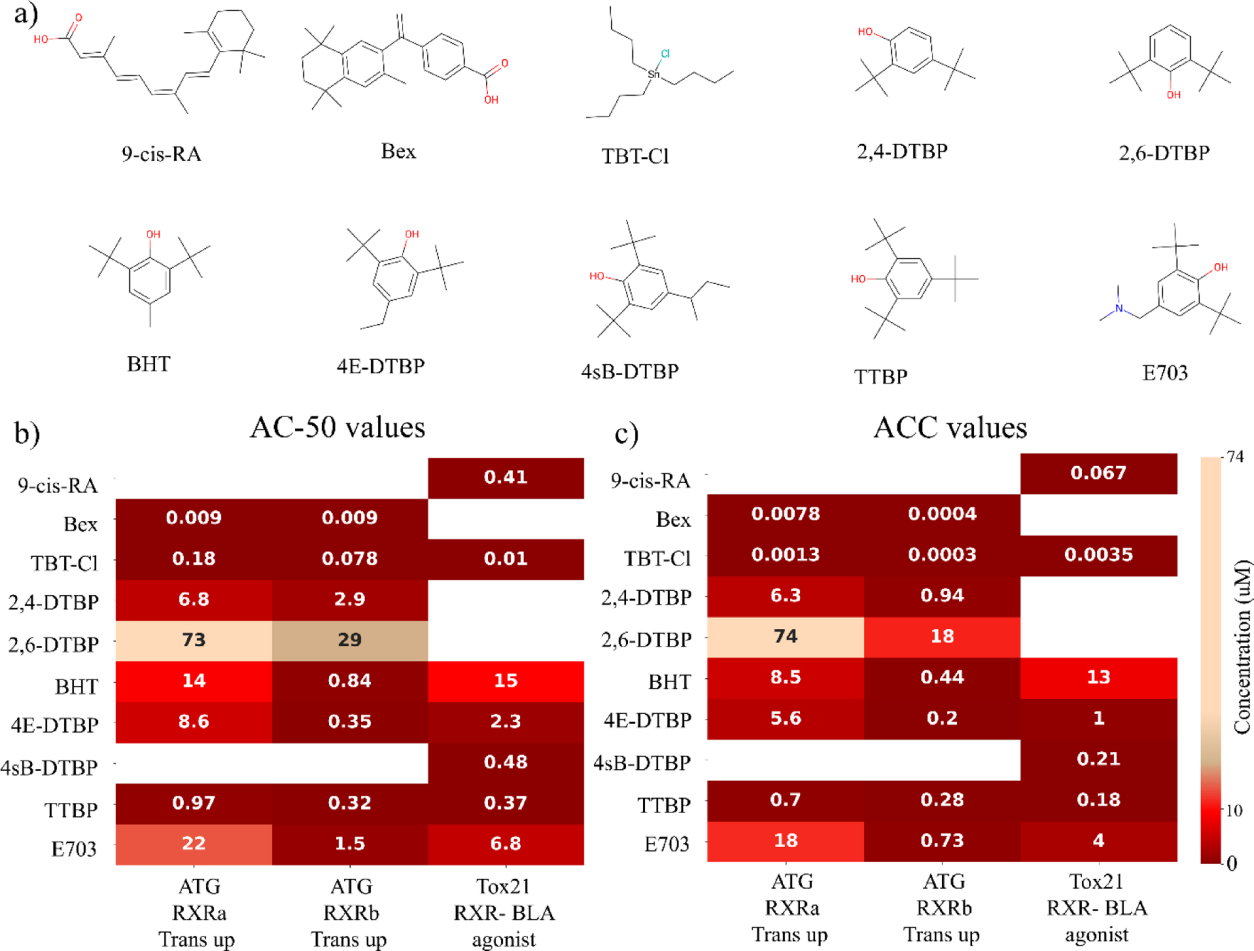
(**a**) Structures of chemicals tested in Tox21 HTS assays. (**b**) Heat map showing the AC-50 (concentration generating 50% activation) in three different Tox21 in vitro RXR activation assays. (**c**) Heat map showing the ACC (activity concentration at statistical cutoff) in the same Tox21 in vitro RXR activation assays. Concentrations are in uM. Blank cells indicate data is not available for this chemical in the given assay. Abbreviations are shown in Table 2.

Due to the potential for human exposure to food contact chemicals and the regulatory interest around them, we selected 5 of these chemicals from Cluster 1 for further evaluation using publicly available high throughput screening assays as well as follow up in vivo assays (Table 1 and 2). Selections were based on the docking score, MM-PBSA free energy, and ML probability (see Supplementary Information, Table S1). As a negative control, we also selected 2,6-DTBP, another tert-butylphenol chemical (Table 1 and 2). 2,6-DTBP is predicted to be inactive by ML and the docking results predict it is a weak binder despite the fact that its binding mode to RXR is similar to the other 5 selected chemicals, being comprised predominantly of hydrophobic interactions (see Supplementary Information Figure S6). The six tert-butylphenols selected for subsequent analysis vary only at the C4 position of the 2,6-di-tert-butylphenol backbone, ranging from a hydrogen (2,6-DTBP) through increasingly bulkier aliphatic groups (4sB-DTBP and TTBP having sec-butyl and tert-butyl groups, respectively) to a tertiary amine (E703) (Fig. 2a).

**Table 2:** MM-PBSA binding free energies and their energy components for different chemical–RXR complexes.

DTXSID	ΔE_vdW_ (kcal/mol)	ΔE_elec_ (kcal/mol)	ΔG_PB_ (kcal/mol)	ΔG_SASA_ (kcal/mol)	ΔG_gas_ (kcal/mol)	ΔG_solv_ (kcal/mol)	–TΔS (kcal/mol)	ΔG_bind_ (kcal/mol)
4sB-DTBP DTXSID8029315	− 42.6 ± 0.22	0.09 ± 0.13	11.25 ± 0.2	− 4.04 ± 0.01	− 42.51 ± 2.61	7.21 ± 1.98	− 19.95 ± 3.47	− 15.36 ± 3.79
TTBP DTXSID2021311	− 42.83 ± 0.2	− 2.66 ± 0.21	15.48 ± 0.28	− 3.89 ± 0.01	− 45.5 ± 3.01	11.59 ± 2.83	− 19.2 ± 4.03	− 14.7 ± 4.64
4E-DTBP DTXSID0029262	− 40.53 ± 0.17	− 2.54 ± 0.13	17.49 ± 0.16	− 3.61 ± 0.01	− 43.07 ± 2.05	13.88 ± 1.59	− 19.81 ± 3.0	− 9.39 ± 3.66
BHT DTXSID2020216	− 37.4 ± 0.18	− 0.04 ± 0.16	11.77 ± 0.25	− 3.47 ± 0.01	− 37.44 ± 2.75	8.3 ± 2.49	− 19.66 ± 3.35	− 9.48 ± 4.32
2,6-DTBP DTXSID6027052	− 32.76 ± 0.18	− 1.46 ± 0.25	12.67 ± 0.29	− 3.29 ± 0.01	− 34.22 ± 2.94	9.38 ± 2.97	− 17.43 ± 3.92	− 7.41 ± 4.68
E703 DTXSID0044997	− 39.08 ± 0.24	− 79.84 ± 1.06	100.89 ± 1.05	− 4.0 ± 0.01	− 118.92 ± 11.25	96.89 ± 10.57	− 18.17 ± 6.1	− 3.86 ± 8.47

### In vitro, cell-based reporter data from curated Tox21 datasets

We obtained curated, high-throughput screening (cHTS; incorporating analytical QC and technological interference flags) RXR-endpoint transactivation data for the 6 tert-butylphenols in Table 2 found in the Integrated Chemical Environment (ICE; http://ice.ntp.niehs.nih.gov) database^21^. We also downloaded cHTS RXR-endpoint transactivation data for the known endogenous, pharmaceutical and environmental RXR agonists 9-cis retinoic acid (9-cis RA), bexarotene (Bex) and tributyltin (TBT-Cl). We included 2,4-di-tert-butylphenol (2,4-DTBP) in this dataset, as this has been shown to function as an RXR agonist in a human stem cell-based adipogenesis assay^22^ and is both present in, as well as, migrates from food contact materials^23^. Figure 2a shows the structures of the 10 chemicals we evaluated using data from Tox21 cHTS RXR based transactivation assays.

We evaluated transactivation assay data from two different RXR-specific cellular transactivation reporter assays in agonist mode (i.e. no exogenous, known RXR agonist was included). Both reporter assay systems made use of a Gal4-RXR fusion protein in which the DNA-binding domain motif of the yeast transcription factor Gal4 (GBD) was fused in frame to the LBD of RXR, which when agonist bound, can recruit coactivators to activate reporter gene expression. The first assay system employed GeneBLAzer™ technology in HEK293T cells, where the human RXRα-GBD fusion activates beta-lactamase expression with a FRET-based fluorescence readout (Tox21 RXR-BLA agonist, in Fig. 2b,c). The FACTORIAL™ NR assay from Attagene^24,25^ allowed simultaneous assessment of activation of both human RXRα and RXRβ in the HG19 clone of the human hepatocellular cell line HepG2 (ATG RXRa Trans up and ATG RXRb Trans up, respectively, in Fig. 2b,c).

In these assays the known RXR agonists, 9-cis RA, Bex, and TBT-Cl, had nanomolar AC-50 and activity concentration cutoff (ACC) values reflecting their well-established potencies. Similar high potencies (nanomolar ACC and AC50s) were observed for the two food-contact butylphenols that have the largest two aliphatic groups among the 6 we studied, namely sec-butyl (4sB-DTBP) and tert-butyl (TTBP). Among these butylphenols, as the size of the aliphatic group at the C-4 position decreased down to BHT, where the C4 R-group is only a methyl moiety, the AC-50 and ACC values increased (Fig. 2b,c), Similarly, compound E703 with a tertiary amine R-group at C4 demonstrated generally higher AC-50 and ACC values than TTBP and 4sB-DTBP, which were especially notable in the RXRα based transactivation assays. Further, our ML model predicted that 2,6-DTBP would be a very poor activator of RXR, and the Tox21 HTS data support that prediction with an ACC value of 18 µM for RXRβ activation, and AC-50 values of 29 µM for RXRβ and 73 µM for RXRα, making it the least potent of all the butylphenols evaluated here. Interestingly, 2,4-DTBP, recently proposed as obesogenic through RXR disruption^22^, had AC-50 values less than 10 µM in the in the FACTORIAL™ NR RXRa and ATG RXRb assays (Fig. 2b). Overall, data from in vitro, cell-based reporter assays for RXR agonism indicate that the most potent RXR agonism by 2,6-tert-butylphenols was observed with those containing butyl groups at the C4 position, with the ability to activate RXR decreasing with decreasing size of the R-group.

### In vivo disruption of a transgenic TR-RXR reporter by tert-butylphenols

Previously, we have used a precocious metamorphosis assay of *X. laevis* tadpoles at 1-week post-fertilization to assess the ability of chemicals to disrupt endocrine signaling through TRs and RXRs^10,11,26^. Metamorphosis is initiated and maintained through the action of THs acting through TRs^14,27^. TRs heterodimerize with RXRs to gain DNA-binding efficiency. Generally, the TR-RXR heterodimer is considered “non-permissive” for RXR agonists, meaning that RXR agonists have no effect on the action of the TR-RXR heterodimer. However, this is not universally true^28^, and we have previously shown that Bex and TBT-Cl can potentiate the action of TRs in young tadpoles through exposure to exogenous T3 before development of the thyroid gland and TH synthesis commences^10,11,26^. Tadpoles at 1-week old are competent to respond to exogenous T3 in many tissues, although they are not able to complete metamorphosis at this stage. We used an integrated luciferase reporter in transgenic *X. laevis* that responds to T3 in tadpole head homogenates at this stage of development^16^ to interrogate the tert-butylphenols predicted by ML to be active RXR ligands and shown by Tox21 cHTS analyses to have RXR agonist activity. We did not include the previously described RXR disruptor 2,4-DTBP^22^, because at 100 nM concentration it caused 100% lethality in less than 24 h. The remaining 6 chemicals we tested did not cause lethality.

**Fig. 3:**
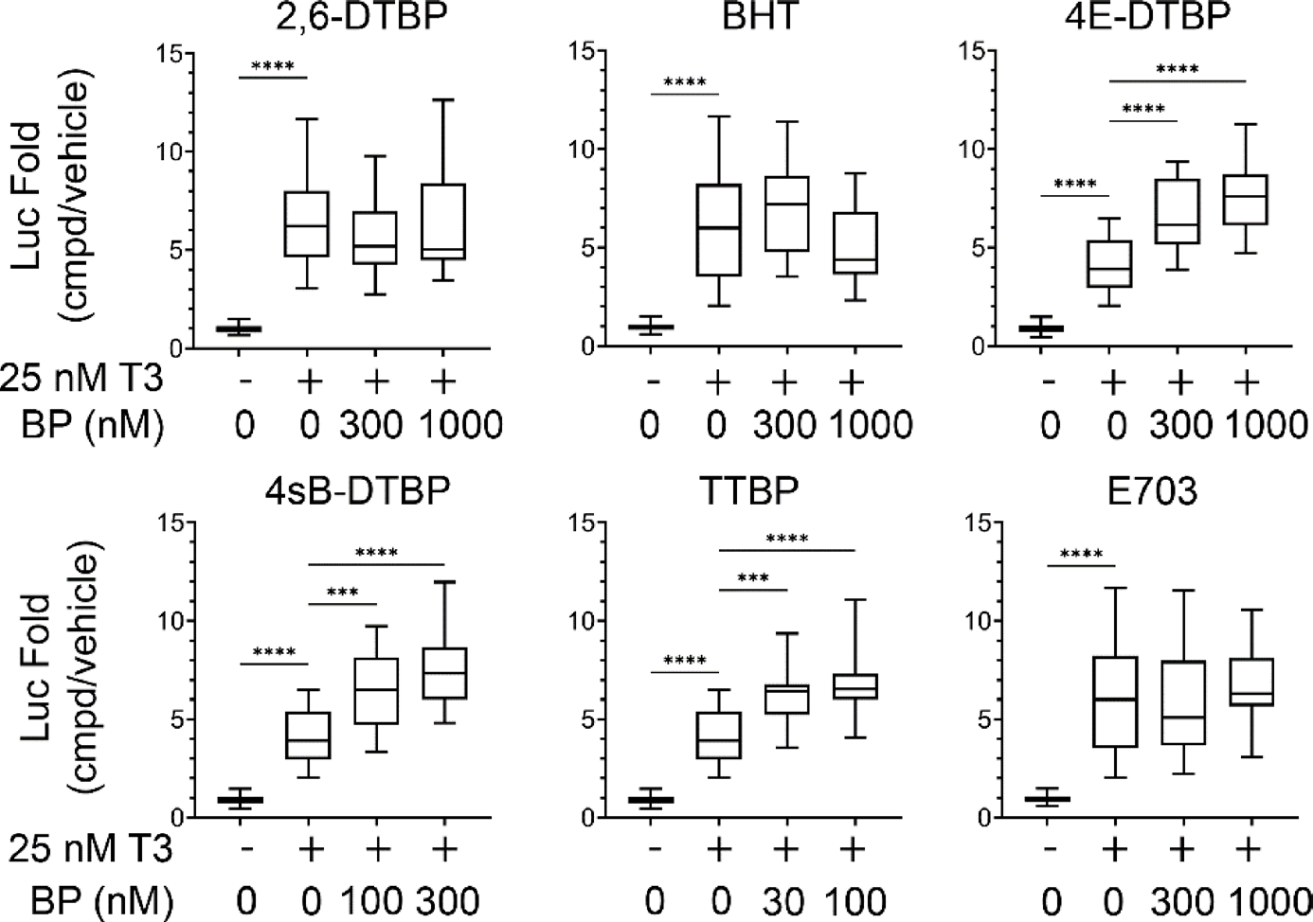
Multiple 2,6-di-tert-butylphenols with aliphatic groups equal to or larger than an ethyl moiety at C4 potentiate T3-activation of an integrated TH-driven luciferase reporter in 1-week-post-fertilization tadpoles. 25 nM T3 caused significant activation compared to vehicle controls, independent of butylphenol status (shown for the T3-only group for clarity). Statistics were calculated as stated in the methods. ***p < 0.001; ****p < 0.0001. Chemical abbreviations are shown in Table 2.

To demonstrate enhanced TR activity by putative RXR agonists, we exposed 1-week old tadpoles for two days to 25 nM T3 and the indicated concentrations of tert-butylphenols through their rearing water (Fig. 3). We use 25 nM T3 in the rearing water to increase the integrated thyroid hormone responsive reporter gene activity during a two day exposure window; tadpoles will survive a five-day exposure to 25 nM T3, even though it is a supraphysiological concentration^16^. Luciferase activity of whole-head homogenates was normalized to total protein concentrations and compared to vehicle controls. T3 significantly activated the luciferase reporter compared to vehicle, independent of butylphenol exposure, but only the significance of chemicals in the presence of T3 compared to T3-alone is shown on the graphs for clarity. Concentrations for butylphenols used in the experiment were chosen by initial range finding and general toxicity exposures as well as the Toxcast in vitro reporter assays. In agreement with inactivity predicted by ML and Tox21 in vitro data, 2,6-DTBP, which has a hydrogen at the C4 position, did not potentiate T3-induction of the reporter (Fig. 3a). At concentrations up to 1 µM, BHT, which has a methyl group at C4, also did not potentiate T3-action (Fig. 3b,f). The increase in size from a methyl to ethyl group at C4 allowed 4E-DTBP to significantly potentiate T3 activation at 300 and 1000 nM (Fig. 3c). Further increasing the size of the C4 moiety to two different forms of butyl groups amplified the ability to potentiate T3-action as 4sB-DTBP treatment resulted at significant potentiation at 100 nM concentration (Fig. 3d), and 30 nM TTBP treatment resulted in significant potentiation of T3-action (Fig. 3e). However, E703, which has a bulky tertiary amine (dimethylaminomethyl) at C4 rather than a branched alkane, was unable to potentiate T3 signaling at 1000 nM concentration (Fig. 3f).

**Fig. 4:**
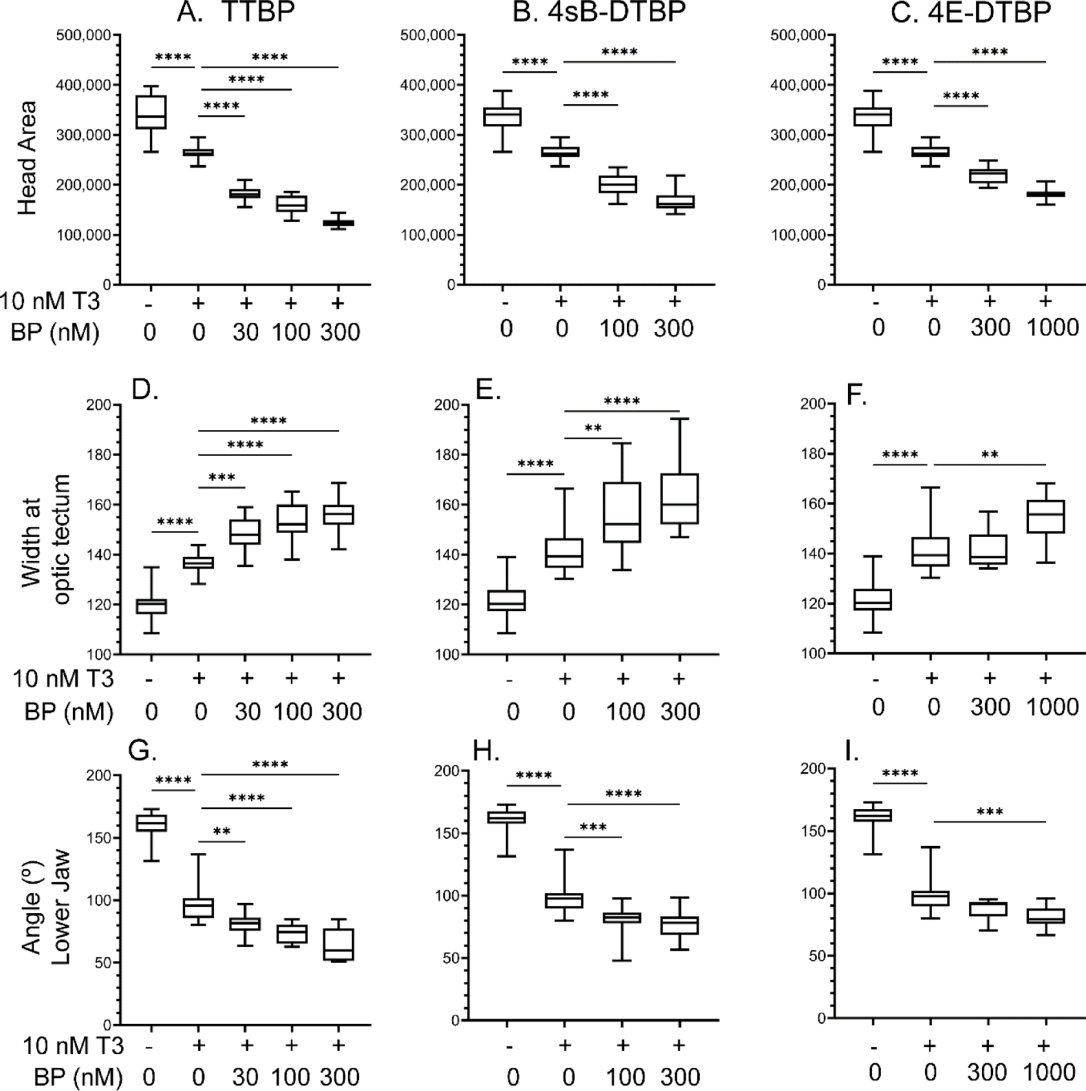
TTBP, 4sB-DTBP, and 4E-DTBP potentiate T3-activated morphological changes in a precocious metamorphosis assay. Measured parameters were Head Area (**A**–**C**); Brain Width at Optic Tectum (**D**–**F**), and Lower Jaw Angle (**G**–**I**). 1-week post-fertilization tadpoles were administered the indicated butylphenols in their rearing water in the presence of 10 nM T3 for five days (TTBP: A, D, G; 4sB-DTBP: B, E, H; 4E-DTBP: C, F, I). Statistics were calculated as stated in the methods. **p < 0.01; ***p < 0.001; ****p < 0.0001. Compound abbreviations are shown in Table 1.

One-week old tadpoles are very uniform in size and morphology, enabling measurement of morphological changes associated with T3 exposure and potentiation of T3 activity by the three tert-butylphenols that were active in the tadpole TR transactivation luciferase reporter. Tadpoles were treated with vehicle, or 10 nM T3 and varying concentrations of tert-butylphenols for five days. 10 nM T3 in the rearing water is a high euthyroid concentration, which we have previously shown allows for gradual changes morphological endpoints over a five-day treatment window^16^. We measured the head area as a proxy for gill resorption^16^ as the majority of the head area consists of the gills, the brain width at the optic tectum, and the angle of lower jaw protrusion from dorsal head photos (see Supplementary Information Figure S8). We note that the tadpole is unable at this early stage of development to remodel the jaw properly^29^; however, the angle of the beak-like protrusion decreases proportionally with increasing T3 concentration to clearly indicate the activation of T3-signaling^11^. The three compounds that failed to potentiate T3 in the tadpole TR-RXR transactivation luciferase assay (2,6-DTBP, BHT, and E703) were unable to potentiate T3 action as assessed by tadpole morphological changes (see Supplementary Information Figure S9). In addition, the three compounds that potentiated T3 in the tadpole TR-RXR luciferase assay (4E-DTBP, 4sB-DTBP, and TTBP) were inactive in the absence of T3 in the morphology assay (see Supplementary Information Figure S10). However, in agreement with our luciferase results, 4E-DTBP, 4sB-DTBP, and TTBP potentiated T3-mediated changes in tadpole morphology in a concentration dependent manner. Indeed, TTBP caused a substantial potentiation of all three morphological changes at 30 nM (Fig. 4a,d,g) with increasing effect to 300 nM TTBP. Figure 4b,e,h shows that 4sB-DTBP could significantly potentiate T3-action across all morphological changes at 100 nM concentration. Identical to our results in the luciferase assay, a minimal C4 moiety of an ethyl group was required to induce significant morphological changes, and that required a 1000 nM concentration to increase the brain width at the optic tectum and to rearrange the lower jaw angle (Fig. 4c,f,i); however, 300 nM 4E-DTBP was sufficient to significantly decrease the head area. In summary, three food contact chemicals TTBP, 4sB-DTBP, and 4E-DTBP were all able to significantly potentiate TH action in an in vivo model for RXR interactions in TH signaling in the nanomolar exposure range. The larger butyl groups at the C4 position were more effective than the smaller ethyl group.

**Fig. 5:**
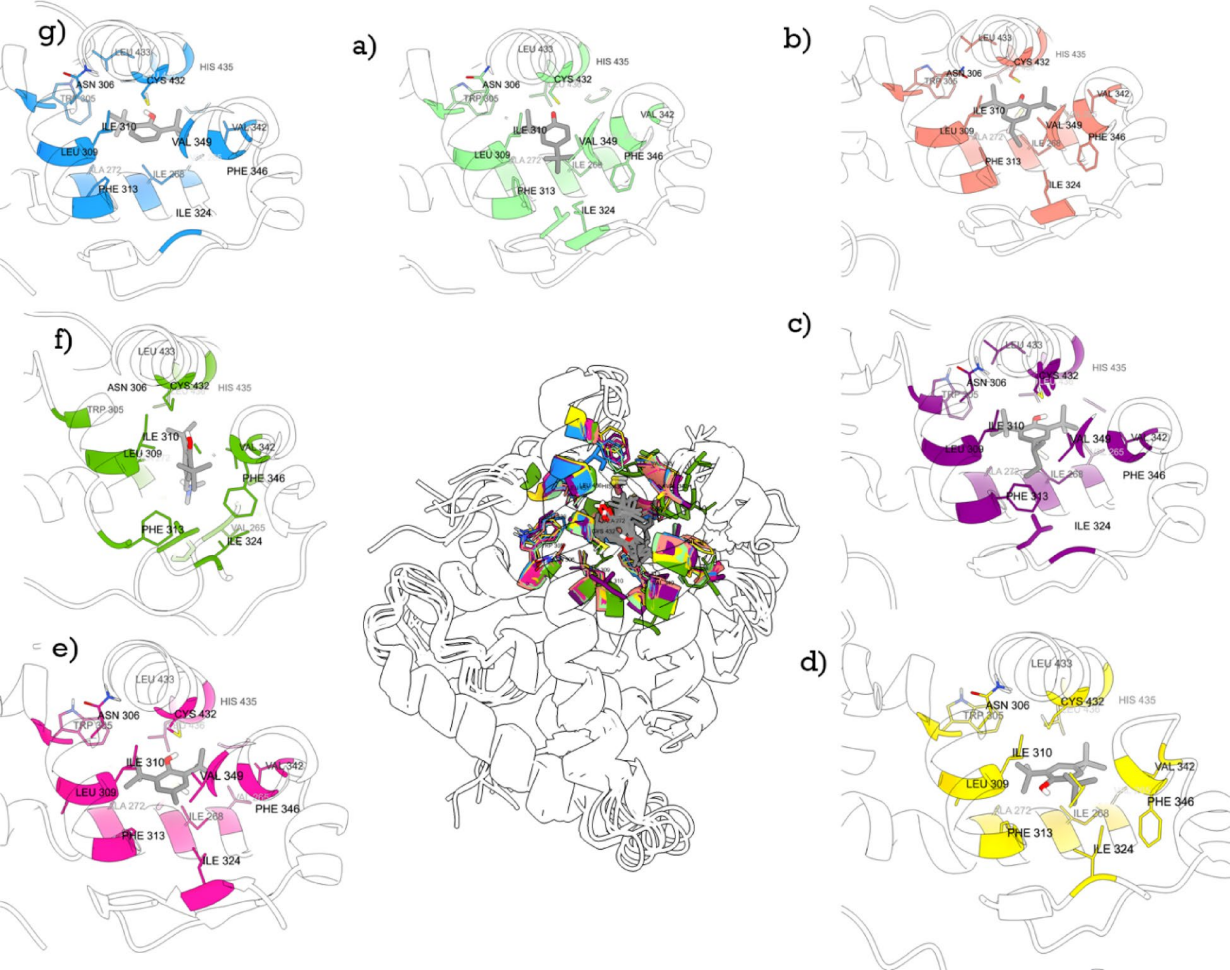
Binding modes of different ligands within the RXRα binding pocket, highlighting key residue interactions. (**a**) 7NKE (crystal structure with 2,4-di-tert-butylphenol), (**b**) DTXSID0029262 (4E-DTBP), (**c**) DTXSID8029315 (4sB-DTBP), (**d**) DTXSID2021311 (TTBP), (**e**) DTXSID2020216 (BHT), (**f**) DTXSID0044997 (E703), and (**g**) DTXSID6027052 (2,6-DTBP).

To understand the molecular-level interactions of these tert-butylphenols with RXR, we performed MD simulation for 100 ns (ns) for each compound using human RXRα (see Supporting information for detail). The binding free energies were calculated for the six tert-butylphenols with RXRα using the MM-PBSA method (Table 2). Among these, TTBP, 4sB-DTBP, and 4E-DTBP showed strong binding affinities characterized by more favorable ΔG_bind_ values (− 14.70, − 17.49, and − 8.48 kcal/mol, respectively). A common feature of these chemicals is their van der Waals (ΔEvdW) interactions with key residues, such as TRP305, LEU309, and PHE313 (Fig. 5 and supplementary information Figure S6). TTBP, 4sB-DTBP, and 4E-DTBP (Figure S6a, b and c) have more frequent, stable interactions across critical amino acid residues, which contribute to stronger binding than other butylphenols. For example, 2,6-DTBP, BHT, and E703 (see supplementary information Figure S6d and S6e) showed weaker and less stable interactions than TTBP, 4sB-DTBP, and 4E-DTBP, particularly with residues critical to activity.

The importance of bulky substituents facilitating these interactions is further supported by the crystal structure of RXRα bound to 2,4-di-tert-butylphenol (PDB: 7NKE)^22^ (Fig. 5a). In this crystal structure, the tert-butyl groups of 2,4-di-tert-butylphenol form extensive van der Waals and hydrophobic interactions within the binding pocket of the RXRα LBD, which are crucial for stabilizing the ligand. Ren^22^ also highlighted that the hydroxyl group of 2,4-di-tert-butylphenol is positioned in close proximity to CYS432 and can adopt two alternative orientations within the binding site.

Similarly, the bulky ethyl, butan-2-yl and tert-butyl at the C4 position of 4E-DTBP, 4sB-DTBP and TTBP (Fig. 5b–d), respectively, appear to enhance steric complementarity within the RXR LBD pocket. This is particularly evident around residues Val349 and Leu309, leading to strong hydrophobic interactions with these residues as well as with Phe313. For TTBP (Fig. 5d), the presence of the third tert-butyl group at the C6 position appears to influence its overall orientation within the binding pocket. Consequently, in our model, the hydroxyl group of TTBP is observed to be positioned more distantly from the Cys432 region compared to the orientation seen in the 2,4-DTBP crystal structure (Fig. 5a) where the hydroxyl directly interacts with the Cys432 vicinity. While our simulations suggest this particular binding orientation for TTBP, we acknowledge that other conformations, potentially involving an interaction of TTBP’s hydroxyl group closer to Cys432 (similar to one of the orientations observed for 2,4-DTBP), might also be possible. The definitive binding mode of 2,4,6-TTBP, including the precise orientation of its hydroxyl group and its interactions with specific residues like Cys432, would best be determined through co-crystallization studies.

In contrast to the strong binders, compounds such as BHT and 2,6-DTBP (Fig. 5e,g), which feature fewer or less effective bulky groups, such as the dimethylamino or smaller methyl groups exhibit fewer and less stable interaction within the hydrophobic pocket. E703 adopts a distinct binding mode (see Fig. 5f) that fails to engage key residues, resulting in weak hydrophobic interactions and reduced steric complementarity, particularly around Val349 and Leu309, as reflected in its lower binding affinity. This results in weak interactions within the hydrophobic pocket and diminished steric complementarity, leading to lower binding affinity as reflected in the free energy calculations. These observations collectively underscore the significant role that the size, nature, and placement of bulky substituents play in modulating the binding affinity of phenolic compounds to RXRα.

## Discussion

In this report we have applied an in silico and ML approach to discover environmental chemicals that can disrupt RXR function in TR signaling. From a starting list of 57,277 chemicals, the NR-Toxpred ML model predicted that 104 chemicals would be active toward RXR. After cluster analysis, we chose six known food contact chemicals for further testing: five tert-butylphenol compounds from the butylphenol class (of 58 predicted positives) and one tert-butylphenol that was predicted to be negative. In vitro Tox21 cHTS data supported the ML predictions for these compounds, with increasing potency with increasing aliphatic bulk at the C4 position of the butylphenol. We tested these six butylphenols in vivo with a developmentally relevant small model organism using an assay suite of a *X. laevis* precocious metamorphosis, where we have shown that RXR disruption disrupts TH signaling; RXR agonists potentiate T3 action and RXR antagonists abrogate T3 signaling^10^. First, we used a transgenic line of *X. laevis* that harbors a single copy of an integrated TH-driven (TH-response element bearing) luciferase reporter. Comparison of T3-activation in the presence and absence of the tert-butylphenols indicated that a minimal ethyl group at the C4 position was required for activation at a 1 uM or below concentration of the tert-butylphenol in the tadpole rearing water. All the active compounds potentiated rather than abrogated T3 action, indicating that they were functioning as RXR agonists not antagonists. Also, 2,6-DTBP, which was predicted to be negative, behaved as expected, and did not potentiate the action of T3 on the reporter. E703, which has a bulky group at C4, did not potentiate T3-action on the reporter; its bulky group contains a tertiary amine that does not appear to be equivalent to the aliphatic groups that were active. Secondly, we quantitatively compared morphological changes induced by T3 signaling in the presence and absence of the tert-butylphenols. Our morphology results agreed with the luciferase data. 4E-DTBP, 4sB-DTBP and TTBP all potentiated T3 action at less than 1 uM concentration in *X. laevis* rearing water.

To further elucidate the structural basis for the above observations, we performed molecular modeling studies to investigate the binding modes of active and inactive tert-butylphenols. 4E-DTBP, 4sB-DTBP, and TTBP share the molecular structure common among congeners of a phenolic ring substituted with ortho-positioned bulky alkyl groups. The binding modes of the active and inactive compounds highlight that the bulky groups of RXR-active tert-butylphenols are located near key residues, particularly Val349 and Leu309. The combination of these hydrophobic interactions and the presence of the bulky groups increases the molecular fit and stabilization within the RXR LBD, resulting in strong receptor activation. The inactive compounds E703, BHT, and 2,6-DTBP, in contrast, had less favorable ΔG_bind_ values (− 3.85, − 9.91, and − 7.45 kcal/mol, respectively). Additionally, the absence of bulky groups in inactive compounds results in a poor fit within the binding pocket, reducing steric complementarity and leaving the hydrophobic pocket inadequately filled. These compounds display weaker or less numerous interactions with the RXR LBD, particularly lacking in significant van der Waals forces as indicated by their lower ΔE_vdW_ values. The network diagrams reveal that these compounds lack critical interactions with the key residues TRP305, PHE313, and ALA272 (which forms the hydrophobic tunnel) that are essential for strong binding of ligands to the RXR LBD^30^. These computational results align with our in vivo findings, where only compounds with favorable binding profiles potentiated T3 activity.

4E-DTBP, 4sB-DTBP, and TTBP are synthetic phenolic antioxidants (SPAs)^31,32^, which are widely used in plastics. Both 4sB-DTBP and TTBP have high production volumes of 100–1000 tons/year, while 4E-DTBP does not have a registered tonnage. According to the Food Contact Chemicals Database, all three compounds are used in food contact materials^9^. Further, TTBP is used in printing inks and children’s car seats; 4sB-DTBP is used in plastics, adhesives, and printing inks; and 4E-DTBP is used in plastics, printing inks, coatings, paper and cardboard packaging, artificial corks, baby bibs, and menstrual products. Because SPAs are additives that are not chemically bonded to substrates, they may migrate out of the substrate and into the foods they contain, the environment, and humans. Indeed, several studies found these three SPAs migrate from food packaging into food simulants^33^. For example, TTBP and 4sB-DTBP migrate into food simulants from packaging, including beverage containers, noodle buckets, and oven and microwave bags and trays^34–36^. Further, 4E-DTBP migrated from food packaging into food simulants and simulated saliva^37–40^. Despite their presence in consumer goods, few studies have evaluated the presence of TTBP, 4sB-DTBP, and particularly 4E-DTBP in environmental and human samples. TTBP was found in household dust in China, Vietnam, Canada, Australia, and the United States^41–44^, and at high levels in sludge from wastewater treatment plants across China^45^. 4sB-DTBP has also been detected in household dust in China, Canada, and the United States^41,44^. 4E-DTBP was not assessed in aforementioned dust and wastewater studies, but has been detected in urban and rural Russian snow and Japanese marine microplastics^46,47^. Although 4E-DTBP, 4sB-DTBP and TTBP are not included in the human biomonitoring programs of the US, Canada, European Union, Korea, or California^48^, 4sB-DTBP has been detected in the gravid plasma, placentas, and breast milk of Chinese women^49,50^ and TTBP has also been detected in the breast milk of Chinese women^50^. Further, TTBP is detected, 4E-DTBP is “expected”, and 4sB-DTBP is not included in the Human Metabolome Database as of its last update in February 2023. These results affirm that humans are exposed to RXR-activating TTBP and 4sB-DTBP, and indicate that humans may be exposed to more butylphenolic compounds, such as 4E-DTBP, than currently realized.

Other studies have shown that RXR disruption can have adverse biological consequences. TBT-Cl was one of the first endocrine disruptors described, causing imposex in farmed oysters, which was later revealed to occur through the activation of mollusk RXR^4,5,51^. TBT-Cl also functions as an obesogen in mice through RXR activation^52,53^. Furthermore, inducing adipogenesis with RXR agonists versus a PPARγ agonist resulted in dysfunctional adipogenesis, wherein the resulting adipocytes expressed more pro-diabetic genes than those induced by the PPARγ agonist^6^. Notably, 2,4-DTBP, a compound of the same chemical class as the compounds in this study, was recently shown to induce adipogenesis in human mesenchymal stem cells through activation of RXR^22^. Their structural analysis also suggested that a broader range of tert-butylphenol compounds could interact with RXRs and affect PPAR signaling via that heterodimer complex, including TTBP. In a transient transfection assay, TTBP was the most potent of the three tested butylphenols both in agreement with our findings and consistent with their structural predictions. In their in vitro adipogenesis assays, the authors used 10 µM 2,4-DTBP, and since 100 nM 2,4-DTBP caused 100% lethality in our tadpoles overnight, we did not pursue this compound. While adipogenesis was not evaluated in our study, the three positive compounds we focused on all potentiated TH action in a precocious metamorphosis model at sub-micromolar concentrations.

TH signaling through TR-RXR is classically considered to be a non-permissive heterodimer, meaning that the ligand for RXR does not play a role in the activating the TR. However, this is not an absolute. For example, high coactivator to corepressor concentrations in pituitary cells can allow the TR-RXR heterodimer to respond to RXR agonists^28^. In addition, in vitro experiments have suggested that RXR agonists help with dissociation of corepressor from the heterodimer, while not recruiting coactivator^54^. Finally, using cellular models, RXRs have been shown to form inactive homotetramers in the absence of agonist, such that little RXR is available for heterodimerizing with its NR partners in the absence of RXR agonist^55^. Any or a combination of these models may be the mechanism through which RXR agonists potentiate T3 action in our precocious metamorphosis model when exogenous RXR ligands are present. We have shown that high concentrations of the pharmaceutical RXR agonist Bex strongly activates the *cyp26* genes which degrade retinoids like 9-cis RA, an endogenous RXR agonist, suggesting that the animal can sense the levels of RXR agonism and tries to compensate for levels that are too high^10^. This compensation, however, fails with non-retinoid RXR agonists, highlighting the potential biological danger of RXR disruption by man-made chemicals.

In summary, we used in silico methods to virtually screen a large library of compounds of environmental interest for their ability to bind to RXRs and disrupt their function. From this primary screen we then tested five tert-butyl phenols predicted with high probability (and one predicted negative) for RXR disruption in an in vivo vertebrate development assay suite that can assess the ability of RXR ligands to disrupt TH signaling. Three of the five (4E-DTBP, 4sB-DTBP, TTBP) were active at nanomolar concentrations, confirming the feasibility of using in silico approaches to predict in vivo binding capacity and biological activity. RXRs interact with one third of the NRs that control early stage body patterning and organogenesis, metabolism, and neurological function, where disruption of RXR function could adversely affect the function of its cognate NR. That these three compounds are used in food contact materials with a major potential for human exposure highlights the need for further evaluation of their toxicological effects and possible regulatory action.

## Methods

### Chemical dataset curation and molecular modeling studies

We expanded the list of screened chemicals from the CoMPARA projects’ original list of 55,450 chemicals, which built on CERAPP’s original list of 32,464 chemicals^56^ compiled from a library of over 50,000 chemicals that humans are potentially exposed to. The main lists included the EPA’s Chemical and Product Categories database^17^, the first version of the EPA’s Distributed Structure-Searchable Toxicity Database (DSSTox)^25,57^, and the Canadian domestic substance list^17^. This library, containing 42,679 chemicals of known organic structures collapsed to 32,464 unique structures known as the CERAPP list after QSAR-ready standardization procedure^58^ and duplicates removal^17^. The CoMPARA list included additional chemicals of interest, as well as simulated ToxCast™ metabolites, such that it totaled 55,450 chemical structures. To this list we added additional substances of interest to the State of California’s Office of Environmental Health Hazard Assessment (OEHHA) and those chemicals listed in the Food Contact Chemical Database (FCCDB), resulting in a total of 57,277 chemical structures. We removed chemicals without SMILES codes and eliminated duplicates based on their Inchi-key. Polymers and inorganic chemicals were excluded.

### NR-Toxpred-machine learning predictions

Initially, we employed the NR-Toxpred machine learning model to predict the activity of the 57,277 chemicals for the RXR receptor^18^. We used an effector model, employing Morgan fingerprints and the SuperLearner algorithm. The applicability domain options were configured with default settings: the minimum number of chemicals, denoted Nmin, was set to 1, and the similarity cutoff Scutoff, was established at 0.25. Predictions within the applicability domain are considered reliable; those outside of the applicability domain may still be reliable, but there is less certainty based on the differences from the training set structures. 104 chemicals were predicted to be active by the NR-Toxpred model that we then subjected to molecular docking calculations.

### Molecular docking of chemicals to RXR receptor

We used an ensemble docking procedure to find the binding poses of chemicals in the ligand binding pocket of the LBD of the RXR receptor. Several structures for RXR were obtained from the Protein Data Bank (PDB), with specific details outlined in Table 3. The selection criteria included factors such as resolution (preference for higher-resolution structures) and diversity in ligand structures (to account for various ligand-binding modes). The crystal structures were prepared by removing crystallographic water molecules, adding hydrogen atoms to the protein, assigning bond orders, and minimizing energy using the protein preparation wizard in the Schrodinger software suite. Using the Glide module in the Schrodinger suite, a grid box of size 10 Å × 10 Å × 10 Å was generated for RXR, centered on the center of mass of the co-crystallized ligand. A curated set of chemicals predicted to be active by the machine learning model was then docked to the ensemble conformations of RXR using the Glide XP algorithm and default Glide settings.

**Table 3:** PDB ID of RXR structures selected for molecular docking with respective resolution and co-crystallized ligand.

Receptor	PDB ID	Resolution (Å)	Co-crystallized Ligand
RXR	1FM6	2.10	(9cis)-retinoic acid
RXR	1FM9	2.10	(9cis)-retinoic acid
RXR	1G5Y	2.00	Retinoic acid
RXR	1K74	2.30	(9cis)-retinoic acid
RXR	1MV9	1.90	Docosa-4,7,10,13,16,19-hexaenoic acid
RXR	1MZN	1.90	4-[2-(5,5,8,8-tetramethyl-5,6,7,8-tetrahydro-naphthalen-2-yl)-[1,3]dioxolan-2-yl]-benzoic acid
RXR	2ACL	2.80	Retinoic acid
RXR	3FUG	2.00	(2E)-3-[4-hydroxy-3-(3,5,5,8,8-pentamethyl-5,6,7,8-tetrahydronaphthalen-2-yl)phenyl]prop-2-enoic acid
RXR	4K4J	2.00	(2E,4E,6Z,8E)-8-(3,4-dihydronaphthalen-1(2H)-ylidene)-3,7-dimethylocta-2,4,6-trienoic acid
RXR	6A5Z	2.95	(9cis)-retinoic acid
MR	2AA2	1.95	Aldosterone
MR	2OAX	2.29	Spironolactone

### Single point MM-PBSA free energy calculation

We used the AMBER 18 software suite to calculate the single point MM-PBSA free energy of binding for each docked RXR-chemical complex^59^. Partial atomic charges for each chemical were calculated using Antechamber with the AM1-BCC method, while the AMBER FF14SB force field was used for the protein. Each complex was solvated using a TIP3P water box and energy minimized in four steps: (1) Minimization of the solute with a restraint weight of 500 kcal/mol/Å^2^ for 1000 steps, (2) Minimization with a restraint weight of 100 kcal/mol/Å^2^ for 1000 steps, (3) Minimization relaxing the solute with a restraint weight of 1 kcal/mol/Å^2^ for 1000 steps, and (4) 2500 steps of steepest descent without any positional restraint. The MM-PBSA binding free energy (ΔGbind) of each minimized complex structure was then calculated using an infinite cutoff (999 Å) and a dielectric protein constant of 4.

### Cluster analysis

A cluster analysis of the chemicals was carried out using principal component analysis (PCA) with Morgan chemical fingerprints as features. K-means clustering was employed to identify distinct clusters within the 102 shortlisted chemicals. The silhouette score was used to determine the optimum number of clusters.

### Molecular dynamic simulation and MM-PBSA Free energy calculation

To gain further insight into the structural dynamics and binding affinity between the shortlisted ligands and RXR, Δ*G*_*bind*_ was calculated using the standard MM-PBSA method implemented in AMBER 18^59^. MD simulations of shortlisted chemicals docked to the RXR were carried out using the AMBER 18 program suite. The docked complexes were solvated with TIP3P water. Using the AMBER14SB force field, javascript:void(0); the solvated system was subjected to 1000 steps of energy minimization employing the steepest descent and conjugate gradient algorithms. Minimization was followed by a preliminary 500 ps MD simulation. The 100 nano second (ns) MD production run was then carried out for all systems using a 2 femto second time step for the integration of the equations of motion in the *NPT* ensemble at 300 K and at 1 atm pressure. The particle-mesh Ewald (PME) method was used to calculate the long-range electrostatic interactions beyond a cutoff of 12 Å. Periodic boundary conditions were applied for all simulations with an isothermal–isobaric (NPT) ensemble at 300 K and 1 atm pressure maintained using the Langevin thermostat and Berendsen weak-coupling algorithm, respectively. The collision frequency was set as 5 ps^–1^. The SHAKE algorithm was used to constrain only hydrogen atoms. The 100 ns production simulations for each ligand/RXR complex were replicated three times, using different random seeds for the initial velocities to get good sampling of coordinate space. The calculated binding free energies calculated using molecular mechanics- Poisson-Boltzmann Surface Area (MM-PBSA) were averaged over the last 50 ns from three different simulations performed with each ligand/RXR complex. The MM-PBSA/GBSA approach was employed to calculate the binding free energy (Δ*G*_*bind*_)) as described previously^60^ to analyze the contributions of electrostatics and van der Waals interactions during the formation of molecular complexes.

### Reagents

All compounds were dissolved in DMSO (Millepore-Sigma, St. Louis, MO). Thyronine (T3) was purchased from Millepore-Sigma (St. Louis, MO) and stored at − 20 C as a 1 mM stock. Tert-butylphenols were purchased from the indicated sources in Table 4 and stored at − 20 C at 20 or 40 mM stocks in DMSO.

**Table 4:** Sources for compounds used in tadpole experiments.

Key	Common name	DTXSID	CAS	Source
26-DTBP	2,6-Di-tert-butylphenol	DTXSID6027052	128-39-2	aablocks (San Diego, CA)
BHT	Butylated hydroxytoluene	DTXSID2020216	128-37-0	Fisher Scientific
4E-DTBP	2,6-Di-tert-butyl-4-ethylphenol	DTXSID0029262	4130-42-1	aablocks (San Diego, CA)
4sB-DTBP	4-(Butan-2-yl)-2,6-di-tert-butylphenol	DTXSID8029315	17540-75-9	Millepore-Sigma
TTBP	2,4,6-Tris(tert-butyl)phenol	DTXSID2021311	732-26-3	Millepore-Sigma
E703	2,6-Di-tert-butyl-4-[(dimethylamino)methyl]phenol	DTXSID0044997	88-27-7	aablocks (San Diego, CA)

### Animal husbandry and tadpole treatments

The natural mating of adult Xenopus laevis frogs and ligand exposure of larval tadpoles were carried out in accordance with an approved University of California Davis Institutional Animal Care and Use Committee (IAUCUC) protocol issued to JDF. UC Davis follows guidelines under full accreditation by the Association for Acreditation of Laboratory Animal Care International (AALAC). Animal experiments were carried out using ARRIVE guidelines (https://arriveguidelines.org/arrive-guidelines). Wildtype, adult, female frogs were primed with 50 IU of either PMSG (pregnant mare serum gonadotropin, Fisher Scientific, Houston, TX) or hCG (human chorionic gonadotropin, Millepore-Sigma, St. Louis, MO) 48–96 h prior to mating, and ovulation was induced with 500 IU of hCG immediately prior to pairing with a transgenic or wildtype male overnight. Males were stimulated with 50 IU of hCG at the time of pairing. Embryo collection and maintenance were performed as described^16^, except that all incubations occurred at RT (approximately 23 C), and animals were incubated in 3-L aquaria starting three days post-fertilization. Treatments were performed as described^10,16^. Treatment concentrations were 10 nM T3, and the indicated amount of test compound. No treatment resulted in increased mortality.

### Tadpole precocious metamorphosis morphology assay

Tadpoles, 1-week post-fertilization (NF 48) were treated with compounds for five days, fixed for photography, and dorsal head shots were taken using a Leica DFC3000 G camera on a Leica MZLFIII Microscope as described^16^. For all morphological measurements, one individual set up the treatments with random placement of tadpoles into the treatment beakers, a second individual who scored the phenotypic outcomes was blinded to the treatments, and measurements were made using the FIJI distribution of ImageJ^61,62^. Head area (proxy for gill resorption), brain width at the optic tectum, and lower jaw angle were measured as described^11,16,26^. A third individual, who had created the blinded treatment groups, then graphed and statistically analyzed the data using GraphPad Prism 10 (GraphPad Software, La Jolla, CA). For statistical analyses, each animal counted as an individual, and at least two clutches (5 tadpoles per clutch) were assayed independently to control for clutch-to-clutch variability. Each clutch was also statistically analyzed independently before combining with other clutches.

### Tadpole luciferase assay

1-week post-fertilization, transgenic tadpoles, expressing luciferase under a TH-driven response element, were treated with the indicated compounds for two days as described^16^. In brief, tadpoles were sorted for GFP-expression in the eye, which co-segregates with the luciferase reporter. Head lysates were prepared as described, and luciferase was measured in a Tecan Infinite M1000 Pro microplate reader using a five second integration time. Luciferase activity was normalized to total protein concentration measured by BCA Assay (Pierce, ThermoFisher, Waltham, MA). Graphing and statistics were the same as for the morphology assay. For statistical analysis, 3–4 clutches (10 animals per clutch) were assayed independently, and each animal counted as an individual. Brown-Forsythe and Welch ANOVAs were used due to significantly different standard deviations among treatment groups. Dunnet’s T3 multiple comparisons test was used to determine significance between treatment groups. All treatments containing 25 nM T3 were significantly activated compared to vehicle controls with p < 0.0001.

## Electronic supplementary material

Below is the link to the electronic supplementary material.

Supplementary Material 1

Supplementary Material 2

Supplementary Material 3

## Data Availability

Data is provided within the manuscript or supplementary information files.
