# Supplementary material for: An in silico to in vivo approach identifies retinoid-X receptor activating tert-butylphenols used in food contact materials
Source: Sci Rep. 2025 Jul 18;15:26102. doi: 10.1038/s41598-025-09244-z (PMC12274580; doi:10.1038/s41598-025-09244-z)
Supplement: Supplementary file 2 — Supplementary Material 2 [file 41598_2025_9244_MOESM2_ESM.pdf]

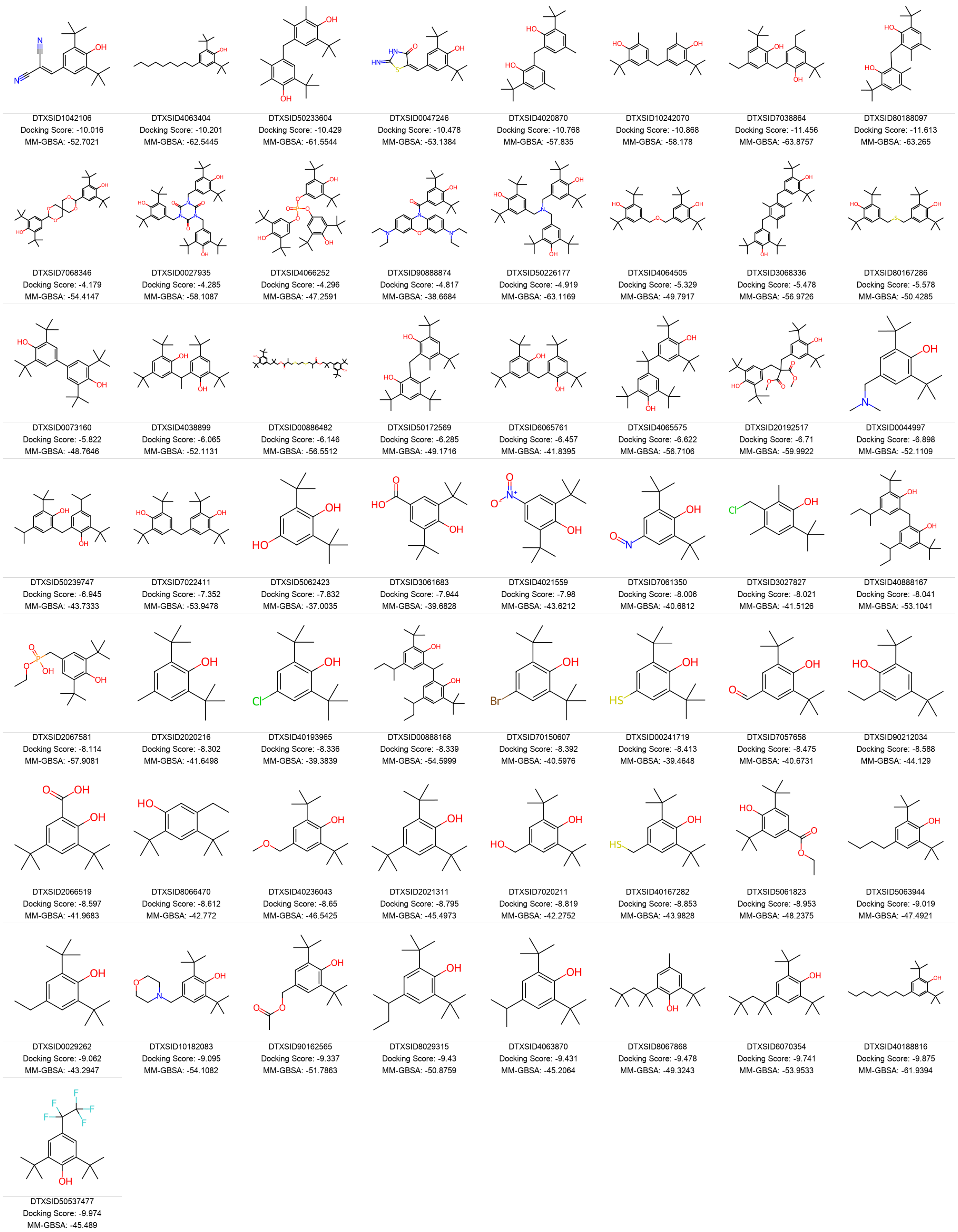

Figure S1 The chemicals in Cluster 1 are the largest group, containing 57 chemicals, primarily phenolic compounds heavily substituted with tert-butyl groups. These chemicals are

characterized by bulky, lipophilic substituents and hydroxyl groups. Cluster 1 chemicals are among the most common food contact chemicals (Groh et al. 2021).

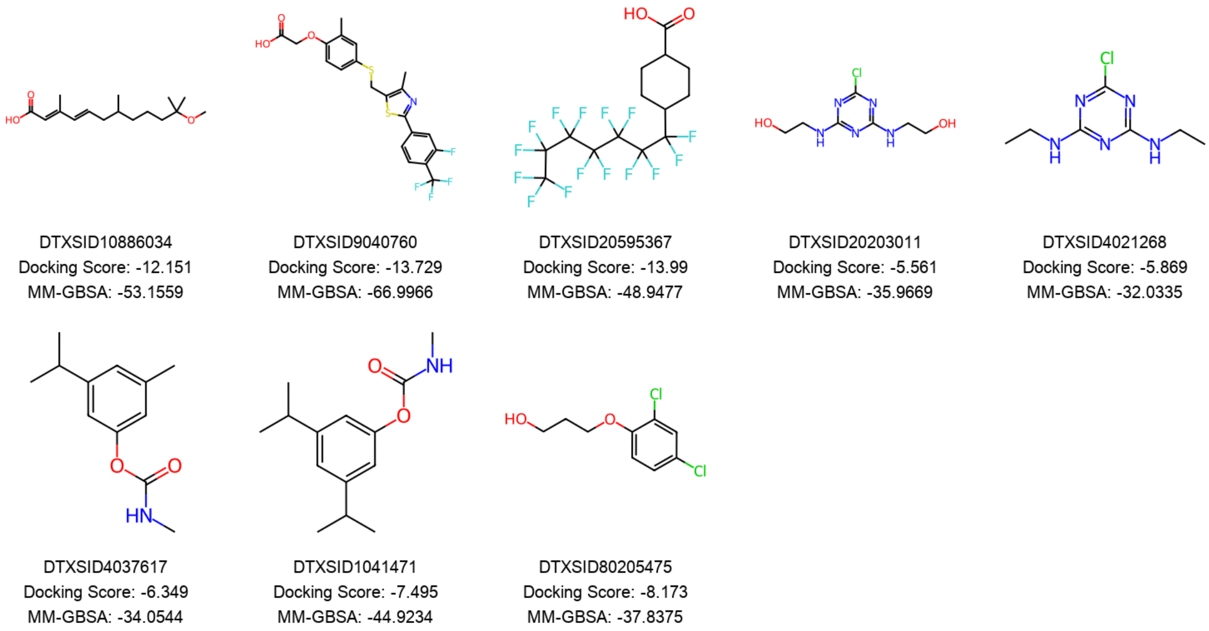

Figure S2 Chemicals in Cluster 2 along with docking score and MM-GBSA free energy. Cluster 2 consists of diverse chemicals with common features including fluorinated groups, heterocyclic rings, chlorinated compounds, and various functional groups such as carboxylic acids, ethers, and esters. The variability in chemical properties and binding strengths is reflected in the significant variability in docking scores and MMGBSA values.

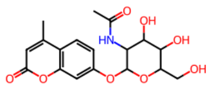

DTXSID20885659
Docking Score: -10.707
MM-GBSA: -55.2279

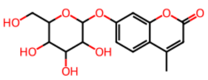

DTXSID30210615
Docking Score: -11.137
MM-GBSA: -49.8117

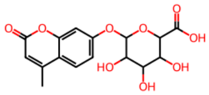

DTXSID10891502
Docking Score: -11.745
MM-GBSA: -58.3063

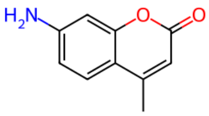

DTXSID40885333
Docking Score: -6.643
MM-GBSA: -32.7138

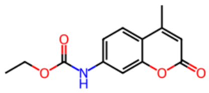

DTXSID3069277
Docking Score: -6.786
MM-GBSA: -37.4954

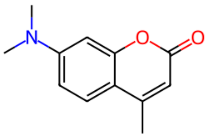

DTXSID6041422
Docking Score: -7.206
MM-GBSA: -40.0196

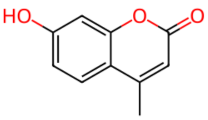

DTXSID1046976
Docking Score: -7.237
MM-GBSA: -32.4975

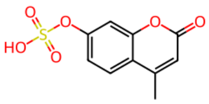

DTXSID8065870
Docking Score: -7.311
MM-GBSA: -46.677

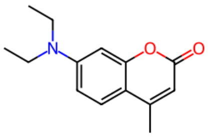

DTXSID9025035
Docking Score: -7.41
MM-GBSA: -42.2305

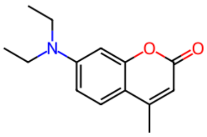

DTXSID40886820
Docking Score: -7.41
MM-GBSA: -42.2305

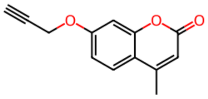

DTXSID3045699
Docking Score: -7.572
MM-GBSA: -37.7759

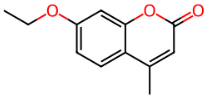

DTXSID3058953
Docking Score: -7.616
MM-GBSA: -36.2711

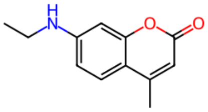

DTXSID1067417
Docking Score: -7.717
MM-GBSA: -38.0087

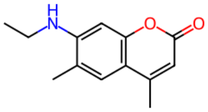

DTXSID20885331
Docking Score: -9.145
MM-GBSA: -34.6832

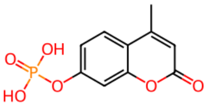

DTXSID3066841
Docking Score: -9.783
MM-GBSA: -43.1685

Figure S3: Chemicals in Cluster 3 along with docking score and MM-GBSA free energy. Cluster 3 includes coumarin derivatives, primarily characterized by the benzopyran-2-one core structure with various substitutions such as methyl, ethyl, amino, and glucuronide groups. This cluster is associated with higher MMGBSA values, indicating weaker binding affinities.

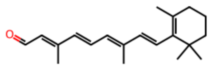

DTXSID0025997
Docking Score: -11.736
MM-GBSA: -60.9607

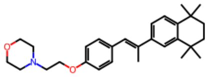

DTXSID7032006
Docking Score: -11.885
MM-GBSA: -71.496

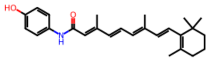

DTXSID2032005
Docking Score: -12.706
MM-GBSA: -67.5119

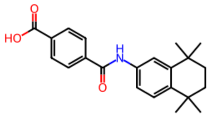

DTXSID5046853
Docking Score: -13.675
MM-GBSA: -62.075

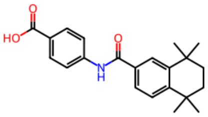

DTXSID5040758
Docking Score: -14.593
MM-GBSA: -64.5511

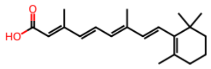

DTXSID4023177
Docking Score: -14.871
MM-GBSA: -62.5631

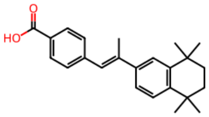

DTXSID6040743
Docking Score: -15.723
MM-GBSA: -69.1099

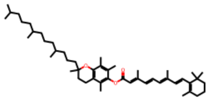

DTXSID6046923
Docking Score: -6.994
MM-GBSA: -69.3417

Figure S4: Chemicals in Cluster 4 along with docking score and MM-GBSA free energy. Cluster 4 comprises retinoid and naphthenyl-derived compounds, including various forms of retinoic acid and its analogs. Many chemicals in this cluster are known RXR ligands with strong binding affinities.

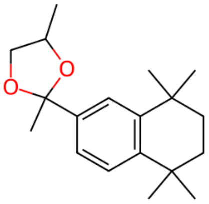

DTXSID00888946
Docking Score: -10.745
MM-GBSA: -54.6808

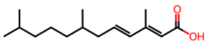

DTXSID00886028
Docking Score: -11.748
MM-GBSA: -49.239

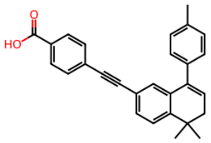

DTXSID5040962
Docking Score: -12.071
MM-GBSA: -55.0321

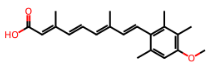

DTXSID6022553
Docking Score: -14.581
MM-GBSA: -66.4457

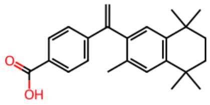

DTXSID1040619
Docking Score: -16.442
MM-GBSA: -69.7711

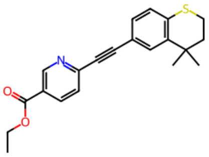

DTXSID5046691
Docking Score: -6.165
MM-GBSA: -56.365

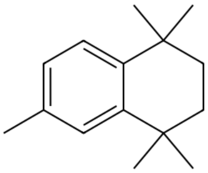

DTXSID50216952
Docking Score: -8.073
MM-GBSA: -39.8246

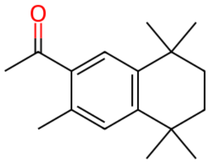

DTXSID7066221
Docking Score: -8.842
MM-GBSA: -44.3628

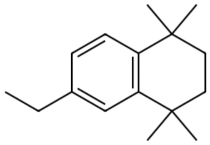

DTXSID7058836
Docking Score: -9.021
MM-GBSA: -41.9898

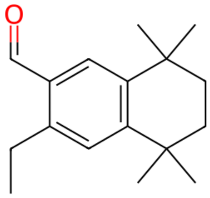

DTXSID7069239
Docking Score: -9.147
MM-GBSA: -41.805

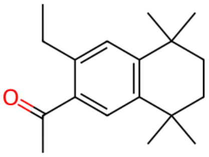

DTXSID3041582
Docking Score: -9.211
MM-GBSA: -48.0481

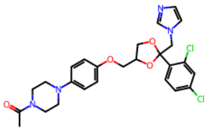

DTXSID7029879
Docking Score: -9.757
MM-GBSA: -77.148

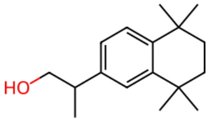

DTXSID40888607
Docking Score: -9.866
MM-GBSA: -47.9454

Figure S5: Chemicals in Cluster 5 along with docking score and MM-GBSA free energy. Cluster 5 contains diverse compounds, prominently featuring naphthalene derivatives and retinoids. Similar to Cluster 4, many chemicals in this cluster are known RXR ligands with strong binding affinities.

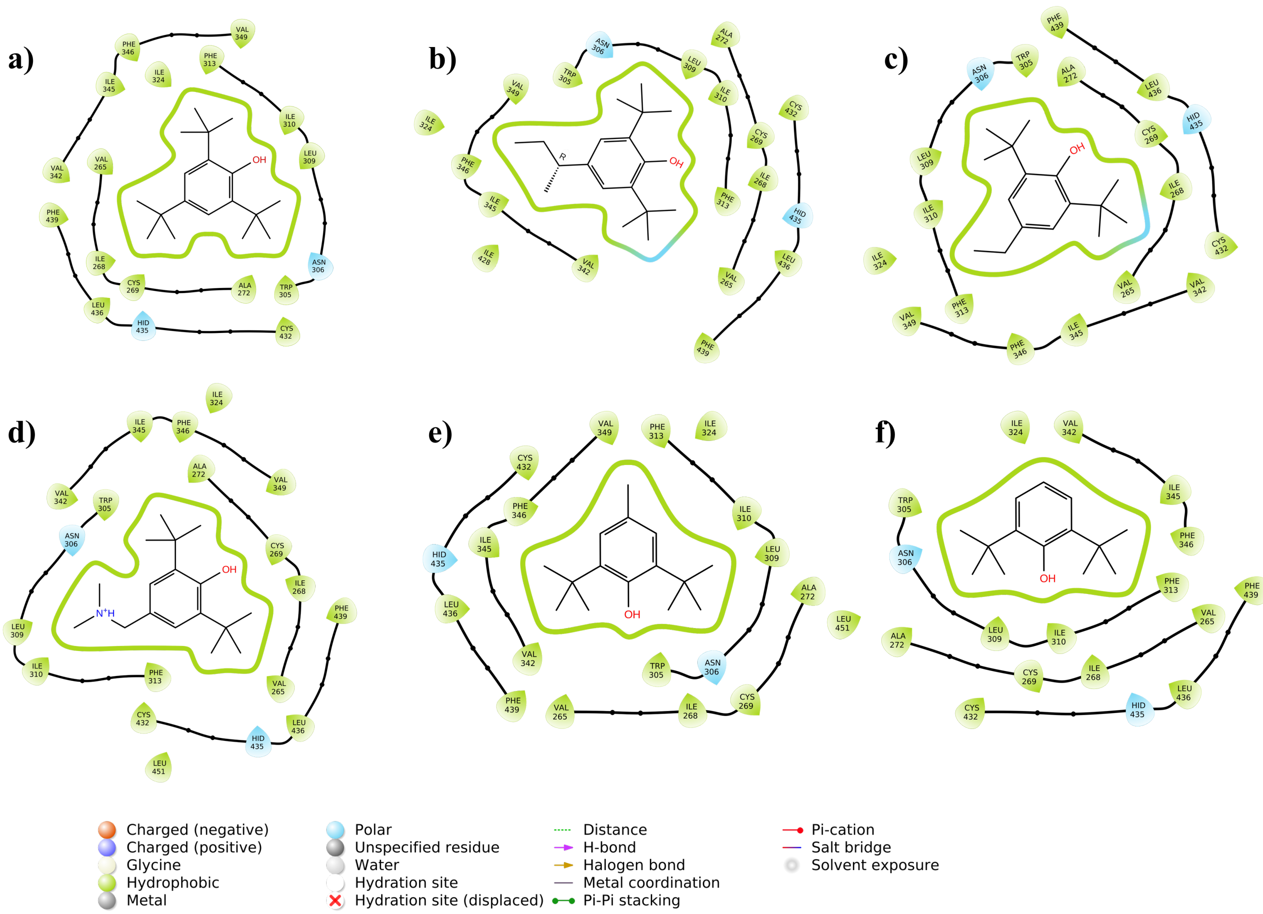

Figure S6. Binding Modes of Chemicals Selected for Further Study: a) DTXSID2021311 b) DTXSID8029315 c) DTXSID0029262 d) DTXSID0044997 e) DTXSID2020216 and f) DTXSID6027052 with RXR Receptor

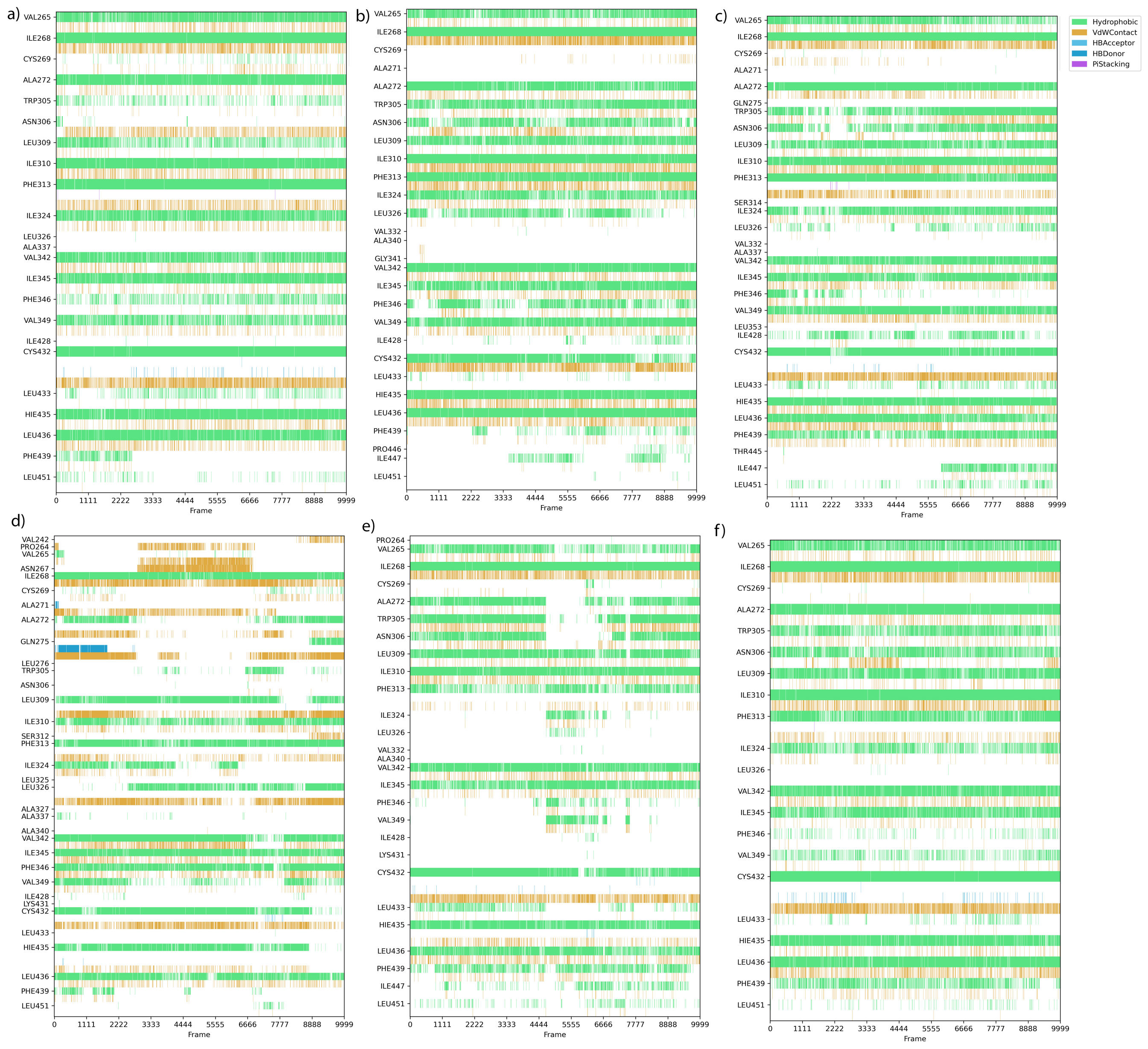

Figure S7: Residue wise interaction of different ligands a) DTXSID0029262, b) DTXSID2021311, c) DTXSID8029315 d) DTXSID0044997, e) DTXSID6027052 and f) DTXSID2020216 with key residues across MD trajectory frames.
