# Supplementary material for: An in silico to in vivo approach identifies retinoid-X receptor activating tert-butylphenols used in food contact materials
Source: Sci Rep. 2025 Jul 18;15:26102. doi: 10.1038/s41598-025-09244-z (PMC12274580; doi:10.1038/s41598-025-09244-z)
Supplement: Supplementary file 3 — Supplementary Material 3 [file 41598_2025_9244_MOESM3_ESM.pdf]

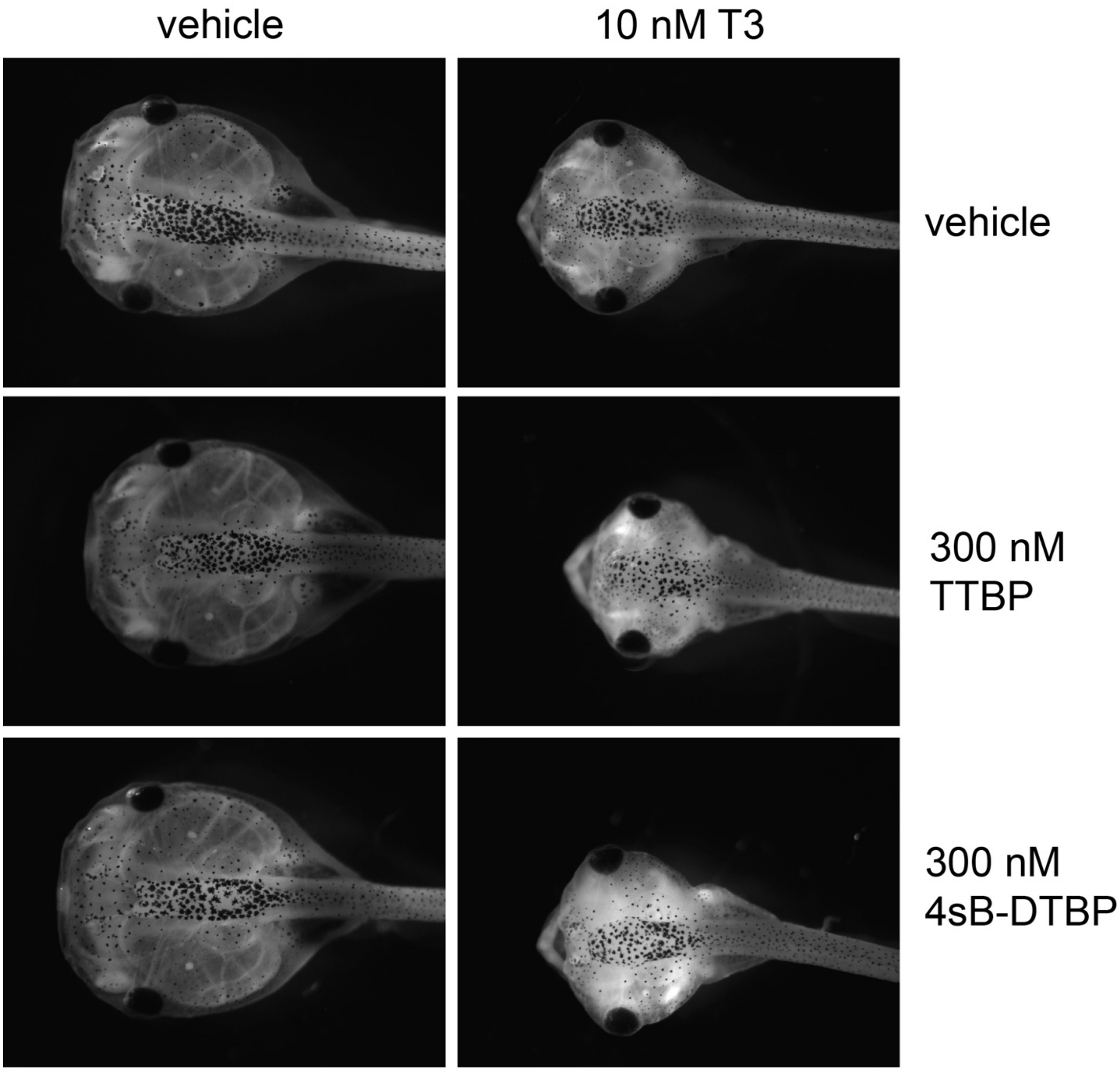

Supplementary Information Figure S8: TTBP and 4sB-DTBP potentiated the effect of T3 on dorsal head morphology, while in the absence of T3, they had no morphological effect.

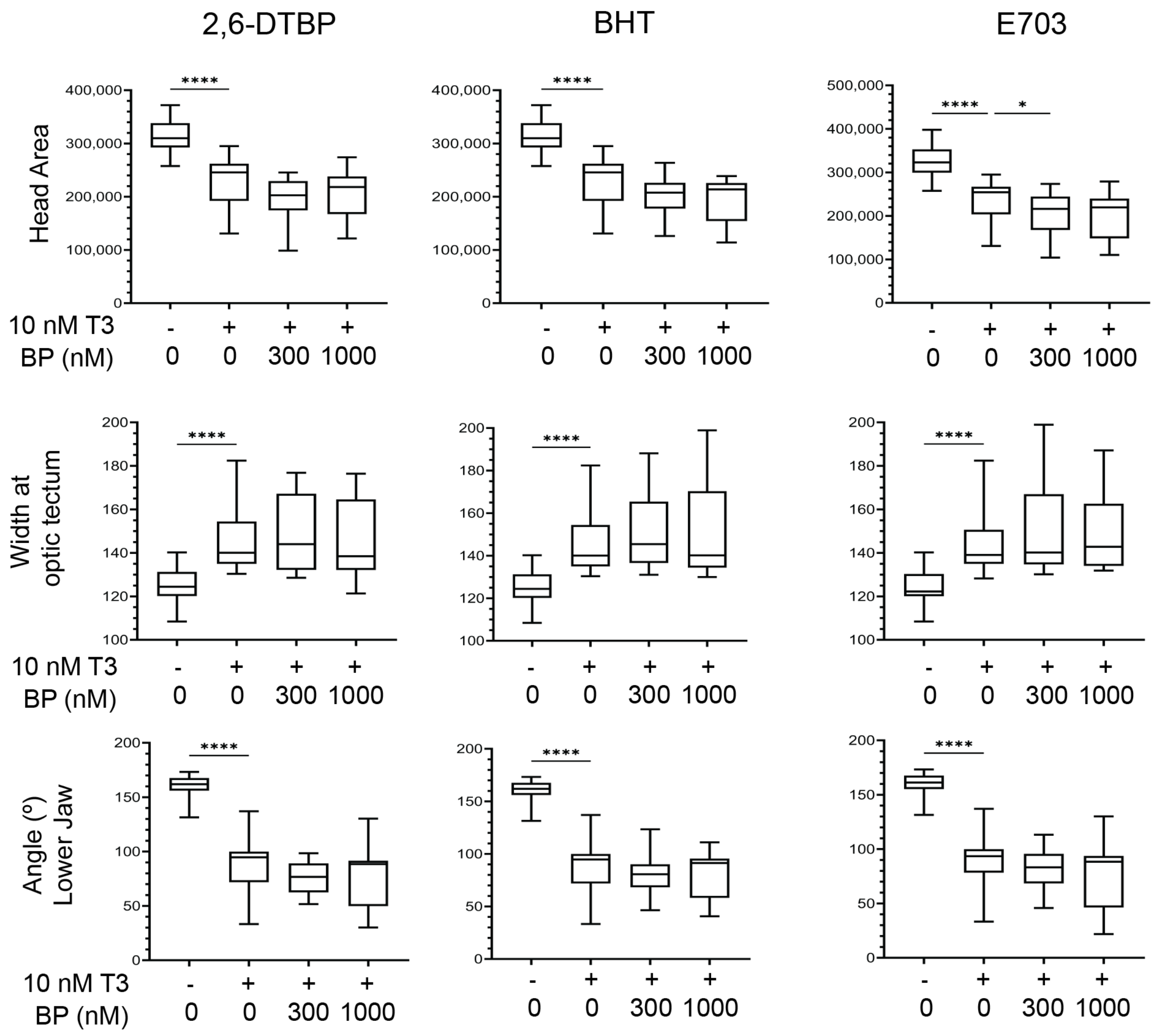

Supplementary Information Figure S9: The tert-butyphenols that did not potentiate T3-action in the luciferase assay were also unable to potentiate T3 in the morphology assay.

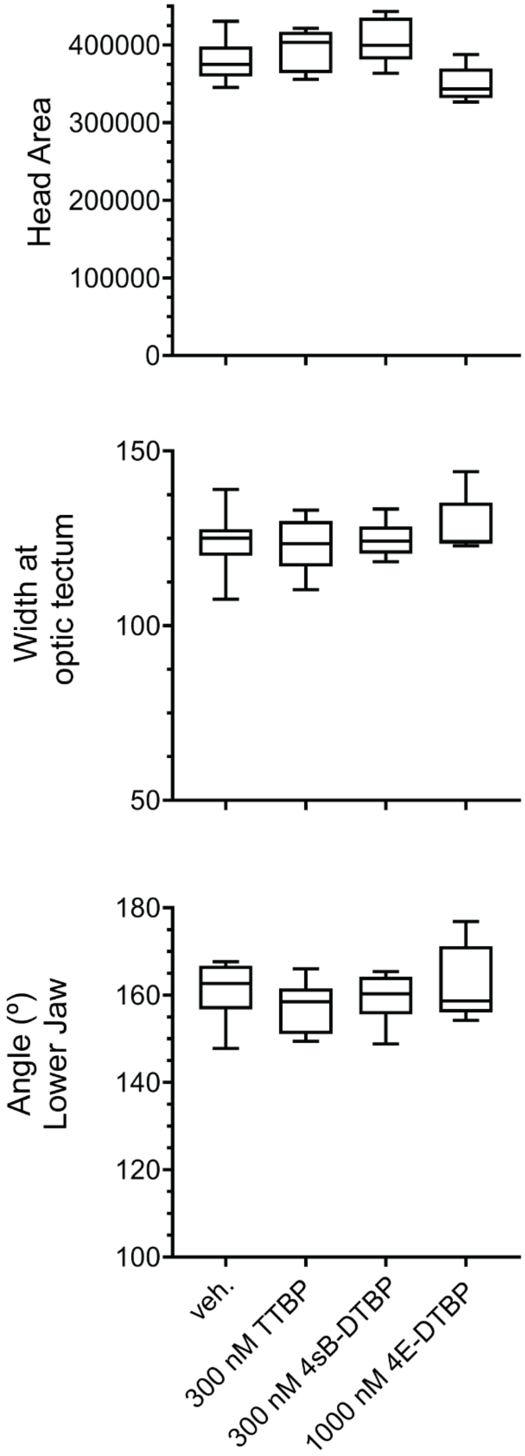

Supplementary Information Figure S10: Tert-butylphenols that potentiated T3 in the luciferase assay were inactive in potentiating morphological changes associated with metamorphosis in the absence of T3.
